# Supplementary material for: Evaluation of a program targeting sports coaches as deliverers of health-promoting messages to at-risk youth: Assessing feasibility using a realist-informed approach
Source: PLoS One. 2020 Sep 2;15(9):e0236812. doi: 10.1371/journal.pone.0236812 (PMC7467304; doi:10.1371/journal.pone.0236812)
Supplement: Attachment — Submitted filename: Reviewer comments PONE-D-20-01877.docx [file pone.0236812.s001.docx]

Reviewer comments PONE-D-20-01877

The authors conducted a process evaluation of an intervention for coaches that work in a Sport-for-development program (SFD). They touch upon a number of interesting aspects in an in-depth case study, describing how various mechanisms play a role in training these coaches for their future work with at-risk youth. As the coaches themselves have a vulnerable background, this offers an interesting view on how interventions like this are perceived and impact the coaches’ behaviour.

Having said that the data is interesting, there are a number of points for improvement. First, I question whether a true realist evaluation has been conducted. It seems that the material does not offer insights on context, mechanisms and outcomes, and how these are linked. Secondly, I would like to urge the authors to offer a more critical perspective on the process, as on several occasions I get the impression that the delivery of the intervention was not optimal and that the impact of the intervention on actual behaviour may have been limited. Thirdly, the authors seem to switch between talking about the mechanisms underlying the training for coaches and the mechanisms of the SFD program that the coaches are implementing for youth. This is very confusing at times, and it leads to conclusions being drawn about the impact of the SFD program that in my opinion cannot be drawn.

Several major and minor issues are elaborated upon below. I hope my comments are received in the same way in which they are intended – as positive and constructive encouragement.

**Major issues**

1. The introduction describes how young people from low SES groups run a higher risk of health issues. In the second paragraph, the authors focus on inequity in access to health services as an important cause in these health differences and how SFD programs can play an important role in prevention of health issues. Although SFD programs can indeed offer a setting in which health promotion efforts can be implemented, whether this also offers potential for reducing health inequities is questionable. I would like to urge the authors to be a bit more careful in and critical towards the potential of SFD programs to reach this. Perhaps it would help to be more specific on what they are investigating: a specific training for SFD coaches to implement health-behaviour messages in a sport program (like it is stated on line 105).

2. The study focuses on the evaluation of an intervention for SFD coaches and what mechanisms underly this training that may impact the coaches’ behaviour. So in my opinion the context is the intervention for coaches and the outcome is the coaches’ behaviour in the SFD program that is given to at-risk youth. However, while reading the introduction, I was confused to see that the authors also talk about the outcome of the SFD program on the health of at-risk youth, which is not the object of study. Alike, in the results and discussion the authors seem to talk about mechanisms underlying the SFD program for at-risk youth, and not the training for the coaches. Perhaps this has to do with the fact that the authors use a lot of anaphora in the text, which makes it difficult to identify what they are referring to. For example on line 563-566, the authors write that “they conducted a sport-for-development intervention targeting coaches with the aim of improving the health of at-risk youth. We were mainly interesting in how the program works...”. Does ‘the program’ relate to the SFD program for at-risk youth or the training for coaches. It would help to be more specific on this and perhaps even omit references to the SFD program for youth from the manuscript. Similarly, ‘impacting the health of at-risk youth’ is not object of study, so perhaps could be deleted in the text to avoid confusion.

3. The authors talk about a realist evaluation, analysing the ‘theory-of-change’, describing context-mechanisms-outcomes, but in reality they are not actually doing a realist evaluation, but rather a ‘normal’ process evaluation, which is also fine given the aim of the study. The training is developed based on a theory-of-change, which is then evaluated by taking notes during the training and interviews/focus group. This does not allow for really falsifying the mechanisms and so I would refrain from making statements about identifying which mechanisms were important or what outcomes were reached. Perhaps it is a suggestion to reflect through the interviews on how these mechanisms were perceived by the coaches. This will be better supported by the data. In this respect, for example on line 462-465 the authors talk about ‘barriers that moderate intervention impact’, but they do not analyse COM configurations.

4. The results section is set up around the mechanisms that were part of the developed intervention. They are called ‘main working elements’ at this point, which adds to the confusion of the reader of what is actually being discussed here. It seems as the authors are discussing how the activities in the coaches’ training are perceived by the coaches themselves and whether the activities actually reflected the mechanisms that were intended by the researchers/developers of the training. I would focus on that, and refrain from presenting data on other aspects, for example how this relates to the SFD program that is implemented for youth and how this impacts these youths’ health.

5. The discussion consists of several sections. The summary (1^st^ paragraph) is not really accurately portraying the results in my opinion. See also my previous remarks about this not being a realist evaluation. The reference to mechanisms of impact and context factors is out of place. They also talk about ‘intervention elements’ (line 570) which is a new term used. The summary of mechanisms is not accurate because some were shown in the results section to be not relevant.

6. The discussion goes on to summarise the different mechanisms as laid out in the change theory, but it does not seem to add something new. These paragraphs could be deleted or become part of the results to explain the mechanisms more in-depth. In addition, on several occasions I felt that the authors draw conclusions that are not supported by the results, or at least they do not seem to directly refer to results that were discussed in the results section. New materials and data are offered here (for example. Lines 588-593: internal motivation was raised OR lines 607-608: about mutual trust). Finally, the authors could be more critical towards the results, because there seems to be drop-out and low dose and fidelity.

7. The conclusion starting at line 738 is too strongly formulated and is not supported by the results. Confusion in use of wording (e.g. employees?, perceived intervention effects etc.). Disparities in dose and fidelity were a strength? I do not agree. A key element was personal health goals, but the authors have found that this was not a working element.

**Minor issues**

1. Abstract: line 35. ‘In their approach..’ This sentence is a big vague and without context. In addition, I am not able to trace this conclusion back to any of the results that I have read.

2. Methods: I would like to see more information about how the intervention was developed. Based on literature, and if so, what literature?

3. Methods: line 134. The mechanisms to promote health of CYP are discussed here, but how are they relevant for mechanisms for the coaches’ training? Why is the results section structured around the mechanisms for health promotion for CYP? (see also point 2, major issues)

4. Page 6, description of intervention. This paragraph is difficult to read. Perhaps a table could help in seeing which sessions were based on which methods and which strategies?

5. Methods line 201-205. Include more information on how notes were taken, based on what? Literature? Check lists?

6. Methods line 207-212. Do the authors know what were major reasons for non-response? Also, I am not sure about the added value of the questionnaires. A scale with only 3 options is not really informative due to the ceiling effect, and they also do not seem to really use the data in the results section (only 2 lines).

7. Results line 281-283. Can the authors reflect on the low dose that was reached? Why was there a need to lower the intensity? Was there no sense of urgency for the coaches. What were the initial goals of the individual sessions? Why was this deemed not necessary anymore? Similar under ‘fidelity’ line 294. Were the take-home assignments not pretested for wording?

8. Results line 296-303. The quote does not seem to reflect the statement ‘participants saw the added value to their work’.

9. The title on line 308 is too bold and does not correctly reflect the data, which describes how the participants perceive the mechanisms and the activities that are implemented in the training to produce these mechanisms. The results do not describe how and when the impact of the intervention is reached.

10. Results line 316-319. Can the authors be more explicit on how ‘the tutor’s openness and willingness to receive feedback’ relates to the ‘coaches/participants motivation’? This seems unrelated. How did the authors see this in the data? In addition, the second quote (line 325) is not really clear to me. I think it misses context to grasp what this person is saying in relation to the analysis of the authors.

11. Results line 329. The line seems to suggest a contradiction with the previous statement on openness, but I do not really see this contradiction. Perhaps, because it is not really clear what the main point is from lines 315-319?

12. Results line 345-347: anecdote is not clear, please specify what is meant with ‘pushing to the limit’ and ‘unconditional’.

13. Quote 376-380. The quote does not seem to address involvement or engagement but rather satisfaction with the program.

14. Page 13 contains three quotes, I think one quote would be sufficient and makes it easier for the readers to see the main point of the authors.

15. Quotes 421-427. Two quotes: to what extent do they address involvement and engagement. I do not really see the link. Moreover, it would be helpful to explicate what the mechanism is that is at work here.

16. Quotes 435-445. Again, one quote will be enough. In addition, the quotes are a bit vague and I do not really read in these quotes the main point that the authors are making in line 429-433.

17. Results 462-466. Results section ‘Barriers that moderate the intervention impact’ is not addressing mechanisms. As this part of the results is directed at identifying mechanisms, is this a relevant point to add? In addition, the authors do not seem to do a Context-Mechanism-Outcome analysis, so they would be unable to identify barriers that moderate the impact.

18. Results section ‘group dynamics and relations’. The authors conclude that this mechanism is not vital, but they do mention it as an element in the abstract. I would leave it out if it is not important. Also, it is confusing that the results section talks about mechanisms, but then also mechanisms that were not playing a role.

19. Results 493-496, is this not more a mechanism of involvement and engagement?

20. Results ‘setting personal health goals’. Can the authors be more explicit that they talk about the health goals of the coaches themselves and not the participants of the SFD program (at-risk youth). I found this confusing throughout the paper. I addition, it is also concluded that this program element did not seem to work. It would be nice if they could offer a more critical analysis of this in the discussion.

21. Results 527-536: this paragraph is vaguely formulated, could the authors provide examples of what they mean for example ‘necessary vocabulary’ and ‘implement new actions in practice’.

22. Quote 542-545: is this quote about the SFD program for at-risk youth? Does the coach say that he keeps track of the emotional tank of the youth? Because that would not fit the ‘mechanisms underlying the training for coaches’. (see also major issue 2).

23. Quote 547-550: I am not sure in what context to place this quote. Who is ‘they’? Is this about the coaches or the at-risk youth?

24. Results 552-554: is this a complete paragraph? What message are the authors trying to get across?

24. Results 556-560: is this not better matching ‘involvement and engagement’.

25. Discussion 637: context elements that impact the intervention. Is this a new topic? Is this also part of the results section, I haven’t seen these. Or are they the same as mechanisms? This is a very short paragraph that needs elaboration. And I wonder if this is actually a result from this current study? Again, no COM configurations were analysed.

26. Discussion paragraph 646-669 could be deleted, does not seem to add anything new.

27. Discussion line 670: authors talk about ‘promising outcomes’ but these were not part of the results section. Further on they talk about data that pointed out that certain factors hamper intervention delivery, but these were marginally discussed and not very thoroughly elaborated upon in the results.

28. Limitations line 711-713: please explain how this influences the findings and their validity. Do the authors feel that valid conclusions can be drawn from the interviews? And to what extent should we be careful drawing conclusions?

29. Limitations line 732-735: paragraph needs elaboration.
